# Editorial: Metabolomics in bacterial infections

**DOI:** 10.3389/fcimb.2025.1557057

**Published:** 2025-02-04

**Authors:** Vijay Soni, Aditya Upadhyay, Yong Zhang

**Affiliations:** ^1^ Division of Infectious Diseases, Weill Department of Medicine, Weill Cornell Medicine, New York, NY, United States; ^2^ Department of Cell and Developmental Biology, Weill Cornell Medicine, Graduate School of Medical Sciences, New York, NY, United States; ^3^ Orthopaedic Soft Tissue Research Program, Hospital for Special Surgery, New York, NY, United States; ^4^ Center of Infectious Diseases and Pathogen Biology, Key Laboratory of Organ Regeneration and Transplantation of The Ministry of Education, State Key Laboratory for Diagnosis and Treatment of Severe Zoonotic Infectious Diseases, The First Hospital of Jilin University, Changchun, China

**Keywords:** metabolomics, host pathogen interaction, metabolism, bacterial metabolomis, system biology

**Figure 1:**
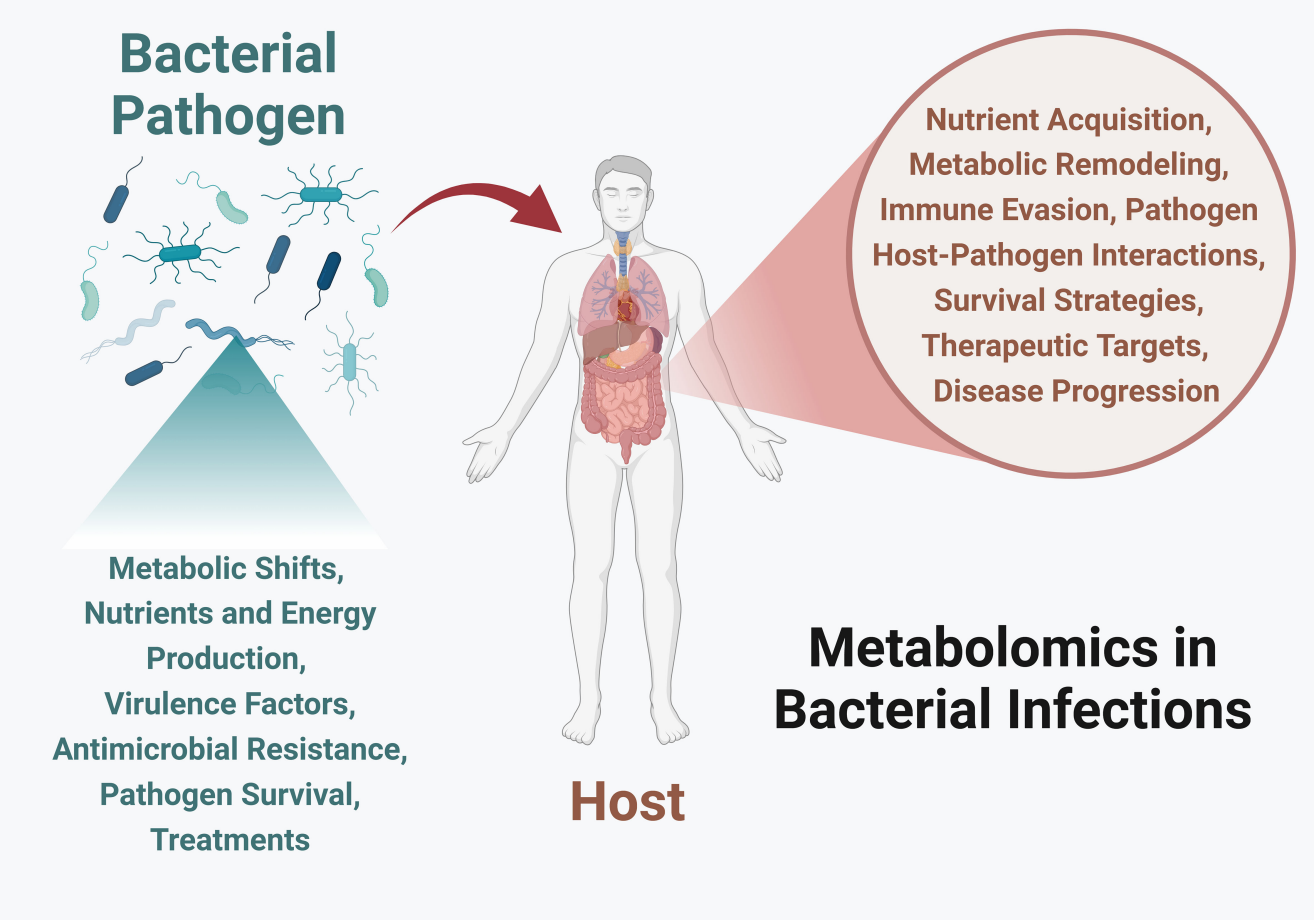
Metabolomics in bacterial infections provides crucial insights into the dynamic interactions between host and pathogens. By studying bacterial metabolic responses, we can understand their nutrient and energy production strategies, and various ways to adapt to host environments, generate virulence factors, evade the immune system, and develop antibiotic resistance. By investigating host metabolism, we can learn about nutrient foraging, changes in metabolic pathways, immune response modulation, pathogen survival strategies, potential therapeutic targets, and the discovery of biomarkers for early detection. Image created using Biorender.

Despite advancements in technology, bacterial infections continue to pose significant global health challenges. Metabolic responses are the first line of changes during infection and represent the best measure to understand the pathogenesis. In this context, metabolomics has emerged as a powerful tool to interpret complex and dynamic biochemical changes. This approach provides valuable insights into bacterial physiology, virulence, and host immune responses, offering new directions for therapeutic development (**Figure 1**). The aim of this Research Topic, *Metabolomics in Bacterial Infections*, was to compile various research articles, methods, and reviews on host-pathogen metabolic interactions, including their modulation and regulation, role in virulence, potential as biomarkers, and promise for novel therapeutic development. We have curated four manuscripts that provide novel insights into host responses to *Coxiella burnetii* infection, metabolic changes during enteric infections and their impact on gut dysbiosis, the genotype-serotype relationship of Group B Streptococcus, and a review of various host-pathogen metabolic changes following *Legionella* infection.

Wan et al. demonstrated that infection with *Coxiella burnetii* upregulates the transcription of the *lyst* gene, which regulates lysosome size and is associated with the pathogen’s effector secretion. LYST limits the expansion of *Coxiella*-containing vacuoles (CCVs) and enhances macrophage immunity by promoting nitric oxide production through the upregulation of *nos2*. These mechanisms help restrict *C. burnetii* replication. Therefore, targeting LYST, by developing small molecule inhibitors, can advance therapeutic strategies against *C. burnetii* and Q fever. However, further investigation into the precise molecular mechanisms involved is needed.

In another interesting study, Hansen et al. employed shotgun metagenomics and untargeted meta-metabolomics to investigate metabolome changes in the human gut microbiome during and after acute enteric infections and recovery. Analyzing data from 60 patients, their findings revealed an increased capacity of microbial metabolic pathways during infection, despite a reduction in taxonomic diversity. Pathways associated with inflammation, such as LPS production, nitrogen metabolism, and glycolysis, were enriched, highlighting the role of Enterobacteriaceae under stress. Post-recovery, a greater diversity of metabolites and the presence of compounds like bile acids indicated a return to homeostasis. Also, these metabolites can distinguish between infected and recovered samples, indicating their potential as biomarkers. The genes involved in these pathways can be further investigated to understand their roles in infection and to aid in the discovery of new drugs.

Further, to understand host-pathogen relation, Zeng et al. used strain-level genomic analysis and assessed the distributions of serotypes and genotypes and their association with virulence genes and host factors (interacting with Srr1 and Srr2) in Group B Streptococcus (GBS). This study highlights the impact of host immunity, strain-specific virulence, and colonization dynamics. Virulence genes such as *srr2*, *hygA*, and *hvgA* are pivotal in GBS hypervirulence, promoting colonization and invasion, while surface proteins like Fbs and Alp help in evading the immune system. The research emphasizes the significance of virulence gene combinations in pathogenicity and suggests that detecting these genes could enhance GBS surveillance, prevention, and vaccine development. However, the study notes the need for broader epidemiological research.

Finally, in a concise mini-review, Wang and Song. explored the metabolic alterations happening in both host and pathogen during *Legionella* infections. They highlighted the role of the bacterial Type IV Secretion System (T4SS) in infection and detailed the roles of key effectors such as MavQ, SidP, and Lgt1-3. These effectors interrupt carbohydrate, lipid, and protein metabolism, manipulate phosphoinositide pathways, and alter membrane properties to inhibit protein biosynthesis, escape immune defenses, and support bacterial growth. The review underscores how understanding *Legionella’s* metabolic interactions with its host can delineate novel drug targets and diagnostic approaches. Despite new discoveries, further studies are required to elucidate these mechanisms across different host systems, paving the way for improved disease management and reduced public health risks.

In conclusion, the studies presented highlight the novel functionality of the *lyst* gene in *C. burnetii* replication, the critical role of the gut microbiome and metabolome during enteric infections, and the intricate relationship between virulence genes, host factors, and metabolic aspects in host-pathogen interactions. These insights can guide novel drug discoveries and serve as biomarkers for disease diagnosis or surveillance. Metabolomics, as a growing field, holds significant promise, and the diverse research contributions in this Research Topic offer new perspectives to combat infectious diseases in a systemic manner.

